# Reassessing the Role of Type II Toxin-Antitoxin Systems in Formation of Escherichia coli Type II Persister Cells

**DOI:** 10.1128/mBio.00640-18

**Published:** 2018-06-12

**Authors:** Frédéric Goormaghtigh, Nathan Fraikin, Marta Putrinš, Thibaut Hallaert, Vasili Hauryliuk, Abel Garcia-Pino, Andreas Sjödin, Sergo Kasvandik, Klas Udekwu, Tanel Tenson, Niilo Kaldalu, Laurence Van Melderen

**Affiliations:** a Cellular and Molecular Microbiology (CM2), Faculté des Sciences, Université Libre de Bruxelles (ULB), Gosselies, Belgium; b Institute of Technology, University of Tartu, Tartu, Estonia; c Department of Molecular Biology, Umeå University, Umeå, Sweden; d Laboratory for Molecular Infection Medicine Sweden (MIMS), Umeå University, Umeå, Sweden; e Division of CBRN Security and Defence, FOI–Swedish Defence Research Agency, Umeå, Sweden; f Department of Chemistry, Computational Life Science Cluster (CLiC), Umeå University, Umeå, Sweden; g Department of Molecular Biosciences, The Wenner-Gren Institute, Stockholm University, Stockholm, Sweden; National Institute of Child Health and Human Development (NICHD)

**Keywords:** RelE, YoeB, ampicillin, single cell

## Abstract

Persistence is a reversible and low-frequency phenomenon allowing a subpopulation of a clonal bacterial population to survive antibiotic treatments. Upon removal of the antibiotic, persister cells resume growth and give rise to viable progeny. Type II toxin-antitoxin (TA) systems were assumed to play a key role in the formation of persister cells in Escherichia coli based on the observation that successive deletions of TA systems decreased persistence frequency. In addition, the model proposed that stochastic fluctuations of (p)ppGpp levels are the basis for triggering activation of TA systems. Cells in which TA systems are activated are thought to enter a dormancy state and therefore survive the antibiotic treatment. Using independently constructed strains and newly designed fluorescent reporters, we reassessed the roles of TA modules in persistence both at the population and single-cell levels. Our data confirm that the deletion of 10 TA systems does not affect persistence to ofloxacin or ampicillin. Moreover, microfluidic experiments performed with a strain reporting the induction of the *yefM-yoeB* TA system allowed the observation of a small number of type II persister cells that resume growth after removal of ampicillin. However, we were unable to establish a correlation between high fluorescence and persistence, since the fluorescence of persister cells was comparable to that of the bulk of the population and none of the cells showing high fluorescence were able to resume growth upon removal of the antibiotic. Altogether, these data show that there is no direct link between induction of TA systems and persistence to antibiotics.

## IMPORTANCE

Within a growing bacterial population, a small subpopulation of cells is able to survive antibiotic treatment by entering a transient state of dormancy referred to as persistence. Persistence is thought to be the cause of relapsing bacterial infections and is a major public health concern. Type II toxin-antitoxin systems are small modules composed of a toxic protein and an antitoxin protein counteracting the toxin activity. These systems were thought to be pivotal players in persistence until recent developments in the field. Our results demonstrate that previous influential reports had technical flaws and that there is no direct link between induction of TA systems and persistence to antibiotics.

## INTRODUCTION

Type II toxin-antitoxin (TA) systems are small operons encoding a toxic protein and an antitoxin protein inhibiting the toxin activity by forming a tight complex (for reviews, see references 1 to 6). The vast majority of toxins are protein synthesis inhibitors using various molecular mechanisms to target different steps of translation (7–11). Antitoxin proteins are labile and degraded by ATP-dependent proteases (i.e., Lon, ClpXP, and ClpAP) (12–14). The expression of TA systems is tightly regulated at the transcriptional level (15–17). In steady-state conditions, the toxin-antitoxin complex acts as a negative transcriptional regulator and binds palindromic sequences located in the operon promoter. Under conditions in which the toxin level is higher than that of the antitoxin, autoregulation is alleviated to restore excess antitoxin.

Type II TA systems are widespread and abundant in bacterial genomes (18–21). TA systems might represent up to 3% of the total predicted open reading frames (ORFs) in some genomes, with some genomes containing more than 90 TA systems. These observations raise essential questions: why are there so many TA systems and what are they for? These questions are mostly unanswered, and the role of chromosomally encoded TA systems in bacterial physiology is highly debated in the field (22–24).

Type II TA systems were first discovered on plasmids in the mid-1980s. Their function in that context is to eliminate daughter cells that did not receive a plasmid copy during cell division and contribute to plasmid maintenance in growing populations (quoted as addiction [25]). In chromosomes, TA systems are mostly part of the accessory genome originating from horizontal gene transfer (20, 26). They are detected on prophages, transposons, and other genomic islands. Their role in such integrated elements is reminiscent of the addiction function (27–29). Other systems are involved in protection against mobile genetic elements such as plasmids (antiaddiction function) or phages (abortive infection) (30, 31). On the basis of their general action on bacterial growth, it was hypothesized that chromosomal TA systems could be integrated in the host regulatory networks and involved in stress management. However, an Escherichia coli strain lacking five TA systems (*relBE*, *yefM-yoeB*, *mazEF*, *chpB*, and *dinJ-yafQ*) for which the toxins are endoribonucleases, had no survival defects in stress conditions (32). In addition, this strain did not show any fitness disadvantage in competition experiments with the wild-type strain. These observations questioned the role of TA systems in stress management. A recent model proposed a direct connection between TA systems and persistence to antibiotics *in vitro*. This model became an instant hit in the field, influencing the research on TA modules and persistence for the last years (33, 34). Persistence is defined as a stochastic switch that pushes bacterial physiology toward an increased antibiotic-tolerant state (35–38). The low frequency (10^−2^ to 10^−6^ depending on the bacterial species, strains, experimental conditions, and antibiotics) combined with the transient nature of persister cells makes them very challenging to study. As a result, the molecular mechanisms underlying persistence remain largely unclear (39). The model linking TA modules and persistence initially stemmed from observations made by the K. Gerdes lab that successive deletions of 10 type II TA systems (later referred to in the field as the Δ10 strain) progressively decreased the level of persistence to antibiotics (33). Deletion of the gene encoding the Lon protease, thought to mediate degradation of different antitoxins, had a similar effect. While this model gained wide acceptance, several independent follow-up studies questioned its validity (40–43). Nevertheless, the model was further refined in a follow-up work that focused on the link between TA system activation and persistence at the single-cell level. The authors reported that stochastic accumulation of (p)ppGpp was the trigger for degradation of antitoxins resulting in activation of TA systems (34). In this work, TA activation was monitored using transcriptional fusions of the *yefM-yoeB* and *relBE* TA operons to *gfp*. The intracellular concentration of the (p)ppGpp alarmone was monitored using a translational fusion between the stationary-phase sigma factor RpoS and mCherry as proxy. Using these reporters, the authors observed that rare nongrowing fluorescent cells within the bulk population of nonfluorescent cells were tolerant to high doses of ampicillin. In some cases, fluorescent cells were able to resume growth after ampicillin treatment. On the basis of these data, they proposed that accumulation of (p)ppGpp inhibits polyphosphatase (encoded by the *ppx* gene), leading to the accumulation of polyphosphate (PolyP). In turn, PolyP binds to Lon and stimulates antitoxin degradation, thereby liberating the toxins from the TA complexes. The resulting free toxins would then inhibit translation and induce persistence. The K. Gerdes lab subsequently proposed that the HipA toxin from the type II *hipBA* system induces persistence through the activation of the 10 TA systems, reinforcing their role as major effectors of bacterial persistence (44).

In a major paradigm shift, the authors of the model discovered that the reference Δ10 strain on which the aforementioned work was performed was severely compromised by infection of φ80 prophages. In their revision, they attributed the observed loss of persistence to these phage infections and disentangled TA systems from persistence (45), leading to the retraction of the two previous papers (46, 47).

Although the notion of a defective Δ10 strain de facto shatters the model, there are additional issues that were not addressed in the revision (45). Given how influential this model has been over the last years, clarifying all these issues remains paramount. It remains unclear how the phage contamination problems would affect the validity of some aspects of the original model, notably the stochastic activation of TA systems in type II persister cells, since these experiments were performed only in the noninfected wild-type strain (34). The same comment holds for the model in which the HipA toxin induces persistence via the activation of the 10 TA systems (44). In this context, we reassessed the roles of type II TA systems by using an independently constructed Δ10 mutant and by testing the fluorescent reporters described in the aforementioned studies. Our results showed that the previously used methodologies have several drawbacks that led to misinterpretation of the results. Besides the highly mutated Δ10 strain, we show that the fluorescent reporters that were used failed to report TA system activation and (p)ppGpp levels. We therefore designed a new fluorescent reporter that monitors induction of the *yefM-yoeB* system at the single-cell level using microfluidic chips coupled with fluorescence microscopy. Interestingly, a small number of type II persister cells were observed; however, fluorescence of these cells was comparable to that of the bulk of the population, confirming that there is no direct link between induction of TA systems and persistence to ampicillin.

## RESULTS

### Deletion of 10 TA systems does not affect persistence to antibiotics.

In parallel to the work performed in the K. Gerdes lab, we constructed a strain with the same 10 TA systems deleted (Δ10LVM) (48). However, the two strains are different in some key aspects. First, the methods used to delete the last five TA operons (see Materials and Methods) in the respective Δ5 strains were different: while we used the λRed method combined with the FLP-FLP recombination target (FRT) recombinase system to remove resistance cassettes from successive deletion mutants (49), the Δ10KG strain was constructed using a counterselection system based on the expression of the type II ParE toxin (33). ParE-based counterselection allowed for scarless deletions but inevitably increased the risk of mutations and rearrangements, since ParE is a DNA gyrase inhibitor (50), which induces DNA double-strand breaks and SOS response (51). Second, while the entire *mazEF* operon is deleted in our strains, only the *mazF* toxic gene is deleted in the strains from the Gerdes lab, allowing the possible expression of the antitoxin *mazE* as well as *mazG*, the third gene of the *mazEFG* operon.

**FIG 1:**
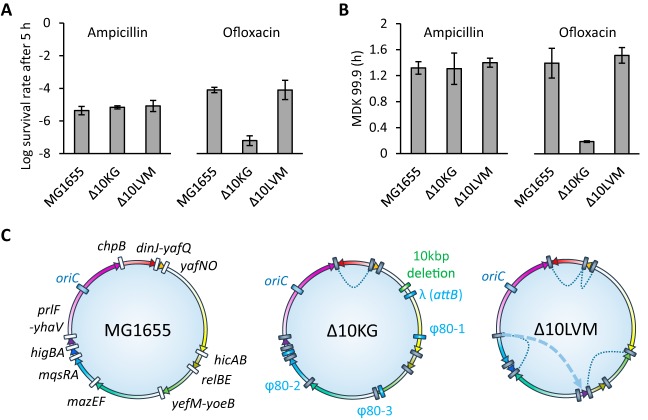
Deletion of 10 type II TA systems has no effect on type II persister cell formation. (A) Surviving fraction of bacteria after 5 h of ampicillin (100 µg/ml) (left) or ofloxacin (5 µg/ml) (right) treatment. Values are the means from at least 3 independent experiments. Error bars indicate standard deviations. (B) Minimum duration for killing (MDK) 99.9% of the population during ampicillin (100 µg/ml) (left) or ofloxacin (5 µg/ml) (right) exposure. Values are the means from at least three independent experiments. Error bars indicate standard deviations. (C) Genome maps of the *E*. *coli* MG1655, Δ10KG, and Δ10LVM strains. Deleted TA loci, phage insertions, and large deletions are annotated in gray, blue, and green, respectively. Colored arrows represent intergenic regions between TA modules in the forward direction. Chromosomal inversions and rearrangements in strain Δ10LVM are represented by dashed lines and arrows, respectively.

Persistence was measured for both Δ10 strains during 5 h of treatment with ampicillin (100 µg/ml) or ofloxacin (5 µg/ml) in steady-state cultures in a chemically defined medium as described in reference 52. The time-kill curves of the different strains have a typical biphasic shape, indicative of a small subpopulation of type II persister cells (see Fig. S1 in the supplemental material). We did not observe any effect on persister formation in the Δ10LVM mutant after either ampicillin or ofloxacin treatment (Fig. 1A). These observations are consistent with a recent correction published by the K. Gerdes lab in which the authors found no effect on persister formation in both ampicillin and ciprofloxacin, in a newly constructed Δ10′TA strain (45). However and as initially reported (33), using the Δ10KG mutant, we observed a 1,000-fold drop in survival to ofloxacin in the Δ10KG mutant. Survival of the Δ10KG mutant to ampicillin was comparable to that of the wild-type and Δ10LVM strains (Fig. 1A). This discrepancy was lately recognized by the authors, as they observed that the difference in persistence to ampicillin between the Δ10 mutant and the wild-type strain could not be reproduced in better defined experimental conditions when chemically defined growth medium was used (45). This supports our assertion that proper and defined experimental conditions are of major importance when performing persistence assays (52).

10.1128/mBio.00640-18.2 FIG S1  Deletion of 10 type II TA systems does not affect growth or sensitivity to ampicillin or ofloxacin. (A) Growth curves of the wild-type strain and Δ10LVM and Δ10KG mutants. Values represent means of two independent replicates. (B and C) MICs of the wild-type strain and Δ10LVM and Δ10KG mutants to ampicillin (B) and ofloxacin (C). Values represent mean values of three independent replicates. Error bars represent standard deviations. (D and E) Killing curves of the wild-type strain and Δ10LVM and Δ10KG mutants to ampicillin (100 µg/ml) (D) and ofloxacin (5 µg/ml) (E). Values represent means of three independent replicates. Error bars represent standard deviations. Dashed lines represent the survival values (10^−3^) MDK was extrapolated from (Fig. 1B). Download FIG S1, PDF file, 0.4 MB. Copyright © 2018 Goormaghtigh et al. 2018 Goormaghtigh et al. This content is distributed under the terms of the Creative Commons Attribution 4.0 International license.

Persistence of these strains was further confirmed by measuring the minimal duration for killing of 99.9% of the population (MDK_99.9_), an accurate parameter to assess survival to antibiotics (38) (Fig. 1B). While 99.9% of the wild-type and Δ10LVM populations were killed by ofloxacin treatment in more than 72 min, this time was drastically reduced to 9 min for the Δ10KG strain. Ampicillin treatment yielded MDK_99.9_ values ranging from 85.2 min for the wild-type strain to 92.4 min for the Δ10KG strain (Fig. 1B). To conclude, the results obtained with an independently constructed Δ10 strain do not support a role for TA systems in persistence and confirms that the earlier report based on the Δ10KG strain is an experimental artifact (45).

### Whole-genome and proteomic analysis of the Δ10LVM and Δ10KG strains.

Whole-genome sequencing was performed on the Δ10KG and Δ10LVM strains, as well as intermediate deletion strains used to construct the Δ10KG strain (Δ5KG, Δ7KG, Δ8KG, and Δ9KG) to help retrace the history of phage infections (Fig. 1C; see Table S1 in the supplemental material). Our analysis confirms that the Δ10KG strain genome is largely rearranged (42, 45). In agreement with the Gerdes lab (45), we found that the Δ5KG strain contains an insertion of a λ prophage at the *attB* site and a φ80 prophage located at the canonical integration site, between *yciI* and *kch* (φ80-1).

10.1128/mBio.00640-18.6 TABLE S1  Genetic rearrangements and polymorphisms identified in TA deletion mutants. Download TABLE S1, PDF file, 0.4 MB. Copyright © 2018 Goormaghtigh et al. 2018 Goormaghtigh et al. This content is distributed under the terms of the Creative Commons Attribution 4.0 International license.

These two phages are detected in all subsequent deletion strains. In addition to these two phages, the Δ7KG and subsequent deletion strains contain another φ80 prophage (φ80-2) located between *glgS* and *ygiJ*. Finally, the Δ10KG strain contains a third φ80 prophage (φ80-3) located between *yeeJ* and *yeeL*. The presence of the three lysogenic φ80 phages in the Δ10KG strain was further confirmed by PCR using specific primers (Fig. S2A). Polylysogen formation by φ80 at these noncanonical sites was previously reported in another context (53). In their recent correction, the K. Gerdes group failed to detect the φ80-2 and φ80-3 phages but identified a φ80-λ hybrid lysogenic phage (45) that we failed to detect. Our data indicate that these phages were progressively acquired during the successive TA deletions, which could be responsible for the progressive drop of persistence observed by the authors during these successive deletions (33). However, we found Δ7KG, Δ8KG, and Δ9KG strains to be genetically identical aside from TA deletions, while the authors showed a progressive drop of survival from the Δ7KG strain to the Δ9KG strain upon antibiotic treatment (33). We thus checked whether ofloxacin treatment induces prophage-dependent lysis of the Δ10KG strain by monitoring turbidity during treatment. We did not observe a drop in turbidity in the Δ10KG culture, suggesting that, despite the 1,000-fold decrease in survival, massive phage-dependent lysis did not occur (Fig. S2).

10.1128/mBio.00640-18.3 FIG S2  Confirmation of phage insertions by PCR and monitoring of bacterial lysis. (A) PCR was used to amplify junctions between attachment sites and prophages to determine the presence of prophages at specific loci determined by whole-genome sequencing. (B and C) Changes in turbidity during ampicillin (100 µg/ml) (B) or ofloxacin (5 µg/ml) (C) treatment. Values represent means of three independent replicates. Error bars represent standard deviations. Download FIG S2, PDF file, 0.4 MB. Copyright © 2018 Goormaghtigh et al. 2018 Goormaghtigh et al. This content is distributed under the terms of the Creative Commons Attribution 4.0 International license.

The Δ10KG strain also contains a 10-kbp deletion encompassing 10 genes. In addition, the Δ5KG strain and its derivatives seem to contain numerous mutations in three of the MG1655 cryptic prophages (DPL12, Rac, and Qin/Kim) as shown by Shan et al. (42). However, reads containing these mutations can also be matched to φ80, suggesting that these polymorphisms might be assembly artifacts due to the presence of φ80 prophages in the Δ10KG strain.

The Δ10LVM strain is devoid of any contaminant prophages (as well as the Δ5LVM strain; data not shown) but shows large chromosomal inversions most likely due to the presence of multiple FRT scars at the deletion sites, allowing for FLP-dependent site-specific and/or homologous recombination between these loci (Fig. 1C). Nevertheless, these rearrangements neither affect growth or sensitivity nor persistence to ampicillin or ofloxacin treatments (Fig. 1A and B and Fig. S1).

We performed label-free quantification mass spectrometry (LFQ-MS) of whole-cell proteomes to compare the Δ10 strains to the wild-type strain (Table S2). In agreement with genomic data, GltI, GltL, and RihA are not detected in the Δ10KG strain, which is deleted for 10 kbp encompassing these genes. The TabA protein level was decreased in the Δ10LVM strain, probably due to a single nucleotide polymorphism (SNP) located upstream of the *tabA* ORF (Table S1). Proteomic analysis also revealed differences in expression of MazG. As mentioned above, in the Δ10KG strain, only *mazF* was deleted, leading to a derepression of the *mazEFG* operon and to higher levels of MazE and MazG (10- and 64-fold, respectively). In the Δ10LVM strain, as expected, MazE and MazG are not detected. It is noteworthy that overexpression of MazG, a nonspecific nucleotide triphosphate pyrophosphohydrolase, has been reported to inhibit growth, prevent (p)ppGpp accumulation, and therefore reduce survival to various stresses (54). However, the persistence rate of a single mutant deleted only for *mazF* is comparable to that of the wild-type strain (33), indicating that overproduction of MazG alone is not responsible for the persistence defect.

10.1128/mBio.00640-18.7 TABLE S2  LFQ-MS analysis of Δ10 strains. Log_2_ LFQ values are represented for proteins differentially expressed in either the Δ10KG or Δ10LVM strain exclusively. Genes associated with proteins represented in bold font also showed genetic alterations in genome sequences. ND, not detected. Download TABLE S2, PDF file, 0.5 MB. Copyright © 2018 Goormaghtigh et al. 2018 Goormaghtigh et al. This content is distributed under the terms of the Creative Commons Attribution 4.0 International license.

### Expression of the *rpoS*-*mcherry* translational fusion is likely to report carryover cells from stationary phase in exponentially growing cultures.

Maisonneuve et al. hypothesized that stochastic synthesis of (p)ppGpp was responsible for toxin activation and growth arrest, therefore contributing to persister formation in exponentially growing cultures (34). In order to test this, they used an RpoS-mCherry translational fusion as a proxy for (p)ppGpp concentration at the single-cell level (34, 44). The authors observed rare fluorescent cells that were persistent to ampicillin, i.e., cells that did not lyse in the presence of ampicillin and were able to resume growth after treatment. They concluded that stochastic induction of (p)ppGpp synthesis leads to persistence to ampicillin. However, the use of an *rpoS* fusion to report (p)ppGpp may be problematic. Regulation of *rpoS* occurs at multiple levels (transcription, translation, degradation, and activity) and involves many regulators besides (p)ppGpp (cAMP, small RNAs, RssB adaptor, ClpXP protease, antiadaptors) (55). Moreover, while (p)ppGpp is involved in basal regulation of *rpoS* expression, it does not appear to play a major role in *rpoS* expression in stationary phase. Strains devoid of (p)ppGpp show full induction of *rpoS* in stationary phase but with a 2- to 3-h delay compared to the wild-type strain (56).

**FIG 2:**
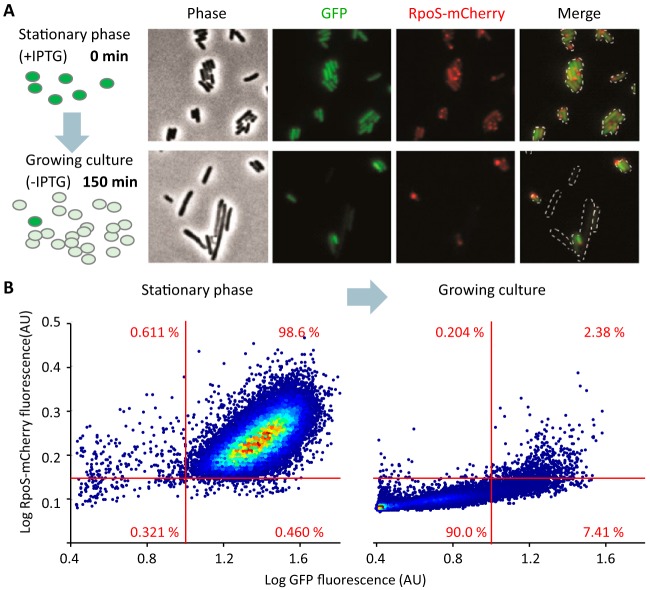
RpoS-mCherry reports nongrowing cells in exponentially growing cultures. (A) Illustration of the GFP dilution system used (57). *E*. *coli* MG1655 *rpoS-mcherry* cells transformed with pET*gfpmut2* were grown to stationary phase with IPTG to induce *gfp* expression, washed, diluted in fresh LB medium without IPTG, and grown for 150 min. GFP will be diluted in dividing cells while it will be retained in nongrowing cells. Fluorescence microscopy images of both stationary-phase and exponential-phase cells are shown. (B) Fluorescence microscopy population analysis of cells prepared as in panel A. A total of 35,185 (stationary phase; left) and 29,469 (exponential phase; right) cells from two independent replicates were identified by CellProfiler. Log median red and green fluorescence values for each cell were measured and plotted. Fluorescence is shown in arbitrary units (AU). The percentage of cells in each quadrant is indicated.

To test the validity of the reporter, we transformed the *rpoS-mcherry* reporter strain constructed by the Gerdes group with a plasmid (pET*gfpmut2*) carrying a *gfp* reporter under control of the inducible p*tac* promoter to monitor proliferation of individual cells (57) (Fig. 2A). Bacteria were grown to stationary phase with isopropyl-β-d-thiogalactopyranoside (IPTG) to induce *gfp* expression, washed, diluted in fresh medium without IPTG, and grown for 150 min to mid-exponential phase, allowing green fluorescent protein (GFP) to be diluted by successive divisions. As expected, most stationary-phase cells displayed both green and red fluorescence. After dilution and growth to exponential phase, both the GFP and RpoS-mCherry fluorescence dropped in the majority of the cells (Fig. 2B). However, some cells (2.38% of the population) retained high red fluorescence concomitantly with high GFP signal, indicating that these cells are carryovers from stationary phase. A small proportion of RpoS-mCherry-positive cells showed no GFP fluorescence (0.20% of the population), indicating that in these cells, *rpoS* might indeed be induced stochastically. Examination of the RpoS-mCherry-positive cells by microscopy showed that, in some cells, the fusion protein was distributed uniformly (Fig. 2A), similarly to the previously published microscopic images (34, 44). However, in many cells, the red fluorescence was localized in dense bodies at the cell poles (Fig. 2A and Fig. S3), which is typical of inclusion bodies and aggregates of misfolded proteins (58). Formation of inclusion bodies suggests that the fusion protein is prone to aggregation and might not be symmetrically distributed during divisions, as previously described for mCherry fusions (59). Thus, the cells with polar RpoS-mCherry signal and low GFP signal in both stationary-phase and growing cultures might be dead or dying cells that have leaked out the soluble GFP but retained the aggregated polar RpoS-mCherry, which accumulated during stationary phase. The inclusion bodies of RpoS-mCherry formed as well when the cells did not contain the *gfp* reporter plasmid (Fig. S3). In addition, we checked that stationary-phase cells of the reporter-free control have no red autofluorescence, showing that the red fluorescence is indeed caused by the production of RpoS-mCherry (Fig. S3).

**FIG 3:**
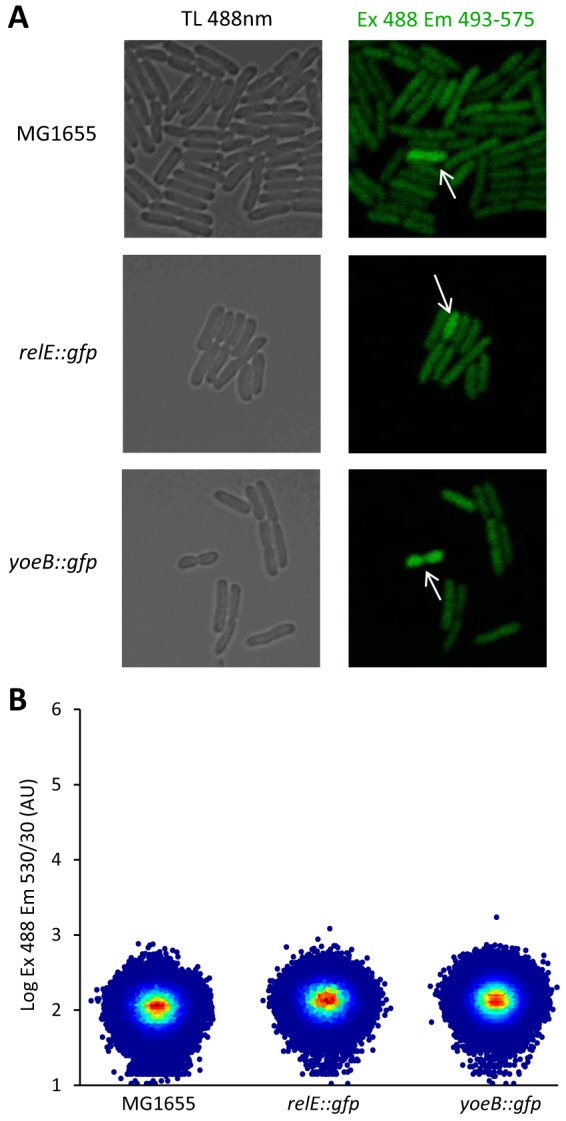
Fluorescence analysis of *TA*::*gfp* reporters. (A) Confocal microscopy of *E*. *coli* MG1655 and its derivatives containing *yoeB*::*gfp* and *relE*::*gfp* grown to exponential phase. The white arrows show cells with above-average fluorescence levels. TL, transmitted light; Ex, excitation; Em, emission. (B) Flow cytometry analysis of strain MG1655 in comparison with the *yoeB*::*gfp* and *relE*::*gfp* reporter strains grown to exponential phase. Analyses were performed on 1,000,000 events. Three independent biological experiments were performed, and a representative example is displayed for each strain.

10.1128/mBio.00640-18.4 FIG S3  RpoS-mCherry aggregates *in vivo*. The plasmid-free MG1655 and MG1655 *rpoS-mcherry* cells were grown to exponential phase and visualized by confocal microscopy as in Fig. 3. TL, transmitted light; Ex, excitation; Em, emission. Download FIG S3, PDF file, 0.3 MB. Copyright © 2018 Goormaghtigh et al. 2018 Goormaghtigh et al. This content is distributed under the terms of the Creative Commons Attribution 4.0 International license.

Altogether, these results indicate that the *rpoS-mcherry* fusion is an inadequate reporter to study formation of persister cells in exponentially growing cultures, as it might report carryover cells from a previous stationary phase instead of stochastic switching to a nongrowing state due to (p)ppGpp fluctuations. However, these results do not rule out a potential role of (p)ppGpp in persister formation. An important step toward answering such question would be the design of a sensitive and specific (p)ppGpp reporter, which to our knowledge, is still missing in the field.

### *TA*::*gfp* transcriptional fusions do not report stochastic activation of toxin-antitoxin transcription.

Stochastic activation of TA modules in type II persister cells became the cornerstone of the model linking the rise in (p)ppGpp levels with the activation of toxins. To test this hypothesis, Maisonneuve et al. monitored the induction of the *yefM-yoeB* and *relBE* TA systems at the single-cell level using transcriptional reporters (34). In their design, the *gfp* gene was inserted downstream of the toxin genes at the TA loci. Green fluorescence was monitored either in microfluidic time-lapse microscopy experiments or in liquid cultures by taking microscopy snapshots. In both setups, the authors found a few cells displaying higher green fluorescence than the bulk of the population. However, the original experiment lacked a necessary control, as the authors did not compare the fluorescence of these strains to that of cells without fluorescent constructs (34). Using the same conditions, we compared the strains carrying the *TA*::*gfp* fusions to the wild-type strain lacking the *gfp* gene (Fig. 3). We were able to detect green fluorescence heterogeneity with confocal microscopy in the *TA*::*gfp*-carrying strains, with some cells being more fluorescent than the bulk of the population. However, we were also able to detect rare fluorescent events that stood out from the rest of the population in the control strain (Fig. 3A). Our results actually show that the fluorescence of these reporter strains is similar to that of a control strain devoid of the reporter constructs. Flow cytometry further revealed that fluorescence distributions are unimodal and similar for the wild-type strain and for both *yoeB*::*gfp* and *relE*::*gfp* reporter strains (Fig. 3B). Moreover, we measured fluorescence using excitation wavelength of 488 nm and recording emission at wavelengths of 530/30 nm and 575/26 nm for the wild-type strain expressing GFP or not expressing GFP and the *TA*::*gfp* strains (data not shown). For the strain expressing GFP, the 530:575 nm ratio is around sixfold. However, the 530:575 nm ratio of the *TA*::*gfp* strains is comparable to that of the wild-type strain, suggesting that the GFP signal of these reporters is weak and masked by autofluorescence in the whole population. The higher autofluorescence of some cells (Fig. 3) may be linked to oxidative stress that has been shown to increase bacterial autofluorescence caused by oxidized forms of riboflavin and flavin nucleotides such as FAD and FMN (60). More severe oxidative damage experienced by some bacteria could explain their nongrowing condition and the nonlysing state during the ampicillin treatment. Interestingly, the nongrowing cells, which had retained RpoS-mCherry at the cell poles, also had a high level of green autofluorescence (Fig. S3). Altogether, these data show that *yoeB*::*gfp* and *relE*::*gfp* reporters do not report expression of the *relBE* and *yefM-yoeB* systems.

### Type II persister cells do not show higher levels of p*yefM*-*yoeB* fluorescence than the bulk of the population.

**FIG 4:**
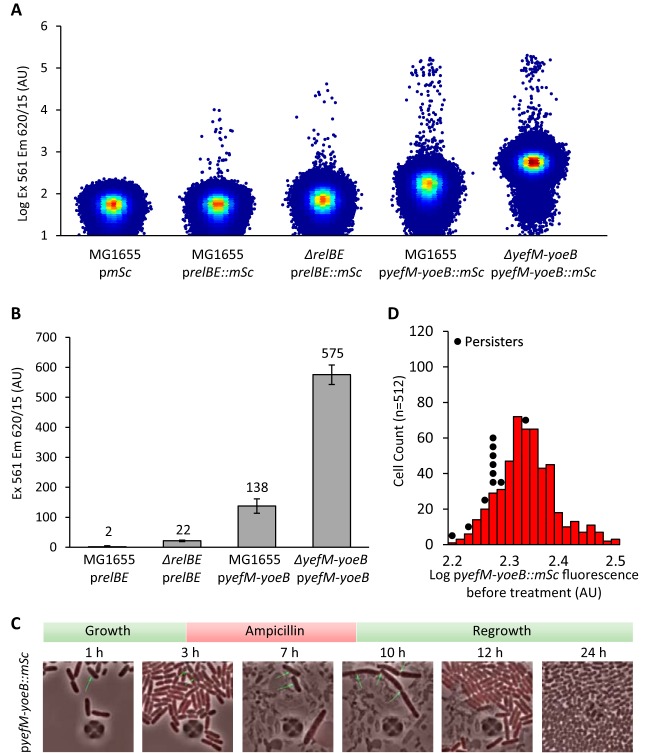
Stochastic expression of *yefM-yoeB* does not lead to persistence. (A) Flow cytometry analysis of cells carrying p*relBE*::*mSc* and p*yefM-yoeB*::*mSc* reporters grown to exponential phase. Three independent biological experiments were performed counting 1,000,000 events, and a representative example is displayed for each strain. (B) Population analysis of p*relBE*::*mSc* and p*yefM-yoeB*::*mSc* expression. Mean population fluorescence values from panel A were corrected for background fluorescence using the mean value of the p*mSc* construct. Error bars represent standard deviations. (C) Time-lapse microscopy of type II persister cells transformed with the p*yefM-yoeB*::*mSc* plasmid. Stationary-phase cells were grown for 3 h perfused in MOPS medium, challenged with ampicillin (100 µg/ml) for 5 h, and regrown for 16 h with fresh medium. (D) Population analysis of p*yefM-yoeB*::*mSc* fluorescence before treatment from Movie S1 in the supplemental material. Fluorescence was measured for 512 nonpersister cells and 11 persister cells. Persisters are plotted above their respective bins as individual black dots.

We thus sought to design more sensitive reporters for TA transcriptional activity. Using a single-copy plasmid, the *relBE* and *yefM*-*yoeB* promoters were cloned upstream of the *mScarlet-I* gene encoding a bright red fluorescent protein (61). Fluorescence of exponentially growing cells containing the p*relBE* and p*yefM*-*yoeB* fusions was analyzed by flow cytometry in the wild-type strain and in the corresponding TA-deleted strains and compared to the wild-type strain containing a promoterless vector as a control. Fluorescence of the wild-type cells containing the p*relBE* reporter is comparable to that of the control (Fig. 4A) with a normalized mean fluorescence of 2 arbitrary units (AU) (Fig. 4B). In the Δ*relBE* mutant, as expected, derepression of the system leads to an 11-fold increase in fluorescence (22 AU [Fig. 4A and B]). For the p*yefM*-*yoeB* reporter, fluorescence of the wild-type cells is substantially higher than that observed for the *relBE* promoter (138 AU), and a fourfold increase in fluorescence is observed in the Δ*yefM*-*yoeB* mutant as a result of promoter derepression (576 AU [Fig. 4A and B]). For both fluorescent reporters, a small subpopulation of highly fluorescent cells is observed, while none was detected with the promoterless fusion, indicating that high fluorescence is specific to these promoters (Fig. 4A and Fig. S4). However, the nature of these cells is still uncertain but does not appear to rely only on TA autoregulation.

10.1128/mBio.00640-18.5 FIG S4  Rare fluorescent events in *TA*::*mSc* constructs. Constructs p*relBE*::*mSc* and p*yefM-yoeB*::*mSc* were transformed in wild-type cells or cells with the corresponding TA system deleted (Δ*relBE*, lower left panel, Δ*yefM-yoeB*, lower right panel), grown to exponential phase, and visualized by fluorescence microscopy. Download FIG S4, PDF file, 0.4 MB. Copyright © 2018 Goormaghtigh et al. 2018 Goormaghtigh et al. This content is distributed under the terms of the Creative Commons Attribution 4.0 International license.

Since the p*yefM-yoeB*::*mScarlet-I* fusion shows detectable fluorescence levels in the wild-type cells, we chose to perform time-lapse fluorescence microscopy in a microfluidic chamber with cells containing this reporter. Among the 2.7 × 10^5^ cells that were analyzed, we could detect 11 type II persister cells (0.0041%) that regrew within 16 h after antibiotic removal (Fig. 4C; see the top panels in Movie S1 in the supplemental material). As far as we know, this is the first direct observation of type II persister cells in wild-type E. coli cells. None of these persister cells showed a fluorescence level above the population average at treatment time (Fig. 4D, black circles). A few highly fluorescent cells (0.012%) were detected and monitored during ampicillin treatment. About half of them (47%) did not lyse but were unable to resume growth after removal of the antibiotic, even 16 h after the end of the treatment (Movie S1, middle panels). Most of these cells showed a significant loss of contrast 16 h after ampicillin removal, indicating damage. The other half lysed during the ampicillin treatment (Movie S1, bottom panels). Altogether, these data show that the type II persister cells we observed did not show a high level of p*yefM-yoeB* fluorescence, underscoring that induction of the *yefM-yoeB* system is not implicated in the generation of persister cells in steady-state growth conditions.

10.1128/mBio.00640-18.10 MOVIE S1  Persistence is not dependent on *yefM-yoeB* induction. Time-lapse microscopy of MG1655 cells transformed with p*yefM-yoeB*::*mSc*. Stationary-phase cells were grown for 3 h perfused in MOPS medium, challenged with ampicillin (100 µg/ml) for 5 h, and regrown for 16 h with fresh medium. The movie shows a combination of persister cells (top panels), fluorescent cells not lysed by the treatment (middle panels), and fluorescent cells lysed by the treatment (bottom panels). Time from the start of the experiment and treatment time frame are shown on the bottom right corner. Numbers in the corner of each series indicate the multiplication of red fluorescence intensity. Figure 4C was sampled from the top left panel. Download MOVIE S1, AVI file, 18.9 MB. Copyright © 2018 Goormaghtigh et al. 2018 Goormaghtigh et al. This content is distributed under the terms of the Creative Commons Attribution 4.0 International license.

## DISCUSSION

The biological role of chromosomally encoded type II TA systems has been extensively debated during the last 25 years. The model linking TA systems and persistence to antibiotics had a major impact in the microbiology community as a whole. Recently, this model was invalidated (46, 47), reopening the question of the role of these widespread systems.

In this work, we provide a series of population and single-cell complementary evidence that further debunk the persistence model previously proposed by the Gerdes group (33, 34, 44). We show here that a newly constructed strain lacking 10 TA systems (Δ10LVM) behaves as the wild-type strain and displays similar persistence levels with ampicillin and ofloxacin. Although this strain contains large genomic inversions, the growth rate, MIC, and persistence rate are comparable to those observed for the wild-type strain. We also constructed transcriptional fusions coupling the promoter of the *relBE* and *yefM-yoeB* systems to the mScarlet-I fluorescent reporter. Fluorescence analysis by flow cytometry showed that the activity of TA promoters is quite low in the wild-type strain, especially in the case of the *relBE* system, as expected due to autoregulation. In the corresponding TA-deleted strain, an increase in fluorescence was observed, therefore validating the constructs.

We used the *yefM-yoeB*::*mScarlet* reporter to monitor TA system induction at the single-cell level using a microfluidic system coupled to fluorescence microscopy. Interestingly, we observed that type II persister cells, those that are able to generate viable progeny after the removal of the antibiotics, did not show high levels of fluorescence. Thus, our work shows that there is no link or role for the induction of the *yefM-yoeB* system in the formation of E. coli persister cells during steady-state growth conditions.

Therefore, the direct outcome of our work reopens a fundamental question involving TA systems: what is the benefit of having so many systems for bacteria? Another important question concerns the redundancy of these TA systems. The persistence model originally arose from the observation that successive deletions of type II TA systems progressively led to a decrease of persistence to both ciprofloxacin and ampicillin. This phenotype was not attributable to any specific systems and led to the erroneous conclusion that TA systems are redundant and have a cumulative effect. Knowing that TA systems are part of the mobilome and are highly variable from one isolate to the other, it appears quite unlikely that they all contribute to a common phenotype. Given the diversity of these systems, their functions might vary depending on their genomic locations, the type of toxin activity, and their bacterial host. One might also consider that they are “just” selfish elements that propagate within bacterial genomes at the expense of their host (22–24).

Several publications implicate (p)ppGpp and type II TA in type II persister formation (11, 62, 63). However, constraining the quite complex phenomenon of antibiotic persistence to a single molecular mechanism or a single genetic cascade is extremely reductive (39). Other (p)ppGpp-independent mechanisms of persister formation implicating factors such as efflux pumps (64), the *tisAB* type I TA system (65), or the concentration of ATP (42) have also been reported. A direct correlation between type II persister cells, (p)ppGpp, and induction of TA systems was considered an alluring prospect driving the field for many years. This assumption was extrapolated from the E. coli and the Δ10KG context and used as the template for research in other bacteria and TA systems. It also gave rise to multiple theoretical models that attempted to simulate and drive conclusions regarding persister cells based on these misguided experimental observations (66–68). Consequently, it remains of paramount importance that such works are reexamined in the light of our results and the current state of the art.

## MATERIALS AND METHODS

### Bacterial strains and plasmids.

Bacterial strains and plasmids used in this study are listed in Table S3 in the supplemental material. The Δ10LVM strain was previously constructed from strain LVM100 (Δ5LVM) (48). mScarlet reporter plasmids were constructed by cloning TA promoters (200 bp upstream of ATG) between AvrII and NsiI sites in pNF02, a derivative of the single-copy plasmid pBeloBAC11. Primers used for the construction of the TA reporters are listed in Table S4. The pNF02 plasmid encodes a codon-optimized *mScarlet-I* transcriptionally insulated by a lambda T1 terminator in 5′ end and a T7 terminator doubled with a two-way LuxIA terminator in 3′ end.

10.1128/mBio.00640-18.8 TABLE S3  Strains and plasmids used in this study. Download TABLE S3, PDF file, 0.4 MB. Copyright © 2018 Goormaghtigh et al. 2018 Goormaghtigh et al. This content is distributed under the terms of the Creative Commons Attribution 4.0 International license.

10.1128/mBio.00640-18.9 TABLE S4  Primers used in this study. Download TABLE S4, PDF file, 0.2 MB. Copyright © 2018 Goormaghtigh et al. 2018 Goormaghtigh et al. This content is distributed under the terms of the Creative Commons Attribution 4.0 International license.

### Media and growth conditions.

Experiments testing *rpoS-mCherry*, *relBE*::*gfp*, and *yefM-yoeB*::*gfp* expression were performed in autoclaved LB to reproduce experimental conditions described in reference 34. All the other experiments were performed in morpholinepropanesulfonic acid (MOPS)-based medium prepared as described in reference 69, supplemented with 0.4% glucose.

### Persistence assays.

Persistence was essentially assayed as described previously (52) with increased sampling frequency. Sampling was performed every 10 min from time zero to 200 min and every 20 min from 200 min to 300 min. Ofloxacin was used at 5 µg/ml, corresponding to 56-fold the MIC for *E*. *coli* MG1655. Ampicillin was used at 100 µg/ml, corresponding to 18-fold the MIC for MG1655. The frequency of persistence is the ratio of the number of colonies at a given time to the number of colonies at treatment time. The minimal duration for killing (MDK) was determined by log linear extrapolation between the two time points bordering 10^−3^ of survival rate to precisely evaluate the minimal time required to eliminate 99.9% of the cells (MDK_99.9_) (see Fig. S1D and E in the supplemental material).

### Whole-genome sequencing.

Genomic DNA was extracted from 2-ml overnight LB cultures using a DNeasy Power Soil extraction kit (Qiagen, Valencia, CA, USA) according to manufacturer’s protocol. The extract was then purified with Agencourt AMPure XP magnetic beads (Beckman Coulter, Beverly, MA, USA) and quantified using the Quantifluor double-stranded DNA (dsDNA) system (Promega, Madison, WI, USA). We sequenced 6 pM genomic DNA (gDNA) on an Illumina MiSeq instrument using the Nextera library preparation protocol and the MiSeq reagent kit v3 (Illumina, San Diego, CA, USA), spiking the flow cell with 1% phiX DNA. Quality of generated paired-end reads were assessed with FastQC and *de novo* assembled using Abyss (70), and obtained contigs were polished using Pilon (71) by mapping reads back to contigs using BWA (72). Generated genome sequences were aligned versus each other using progressiveMauve (73), and structural variants were visualized with genomeRing (74). Single nucleotide polymorphism (SNP) differences were characterized using snippy and dnadiff (75). PHASTER (PHAge Search Tool Enhanced Release) (76) was used to search for potential phage insert in the genome assembly. All genomic analyses were performed using Snakemake (77) as workflow manager together with software installations from Bioconda (78). Reads and assemblies for Δ10KG and Δ10LVM have been deposited in the NCBI BioProject Repository (PRJNA454100).

### Analysis of *rpoS-mCherry* expression.

Cultures of *E*. *coli* MG1655 and MG1655 *rpoS-mcherry* containing the pET*gfpmut2* plasmid were grown overnight in LB medium supplemented with chloramphenicol (25 µg/ml) and 1 mM IPTG to induce *gfp* expression. Bacteria were harvested by centrifugation, washed twice with phosphate-buffered saline (PBS) to remove the traces of IPTG, diluted 100-fold in 10 ml of fresh IPTG-free LB, and incubated at 37°C with shaking in a 100-ml flask. Bacteria were sampled immediately after the dilution and during growth, spotted onto agar pads and imaged with a Zeiss Axio Observer.Z1 microscope equipped with a 63× objective, AuroxCam camera, and filter set 61 HE (Colibri). Cells were detected from phase-contrast images, and the median values of red (mCherry) and green (GFP) fluorescence of each bacterium were measured using Cell Profiler software.

### Fluorescence analysis of chromosomally encoded *relE*::*gfp* and *yoeB*::*gfp* fusions.

Overnight cultures grown in LB medium were diluted 100-fold in 10 ml of LB and incubated at 37°C with shaking in a 100-ml flask. Bacteria were sampled immediately after inoculation and during growth and analyzed using an LSR II (BD Biosystems) flow cytometer equipped with a 488-nm laser, a 530/30 nm filter, and a 575/26 nm filter. Microscopic images of bacteria were acquired using a confocal fluorescence microscope (LSM710; Zeiss). A 488-nm laser and a 493-to-575-nm emission window were used for detection of green fluorescence.

### Single-cell analysis of *yefM-yoeB*::*mSc* expression.

Overnight cultures grown in MOPS medium containing glucose (0.4%) supplemented with Casamino Acids (0.2%) (Difco) (vitamin free) and sodium bicarbonate (10 mM) were diluted to an optical density at 600 nm (OD_600_) of 0.02, grown 3 h to exponential phase (OD_600_ of ~0.3), and diluted in PBS before injection into an Attune NXT flow cytometer. 10^6^ events per experiment were analyzed with a 561-nm laser and a 620/15 emission filter. The same overnight cultures were diluted 50 times in PBS and loaded into a B04A CellASIC ONIX plate. Trapped cells were perfused for 3 h in MOPS medium under 1 lb/in^2^, challenged with the same medium supplemented with ampicillin for 5 h, and regrown with fresh medium for 16 h. Images were taken every 15 min using a Zeiss Axiobserver.Z1 microscope equipped with an ORCA-Flash 4.0 complementary metal oxide semiconductor (CMOS) camera and filter set 00.

Analysis of fluorescence was performed using the MicrobeJ suite for ImageJ.

10.1128/mBio.00640-18.1 TEXT S1  Supplemental Materials and Methods. Download TEXT S1, PDF file, 0.3 MB. Copyright © 2018 Goormaghtigh et al. 2018 Goormaghtigh et al. This content is distributed under the terms of the Creative Commons Attribution 4.0 International license.
