# Supplementary figures and images for: Reassessing the Role of Type II Toxin-Antitoxin Systems in Formation of Escherichia coli Type II Persister Cells
Source: mBio. 2018 Jun 12;9(3):e00640-18. doi: 10.1128/mBio.00640-18 (PMC6016239; doi:10.1128/mBio.00640-18)

**A**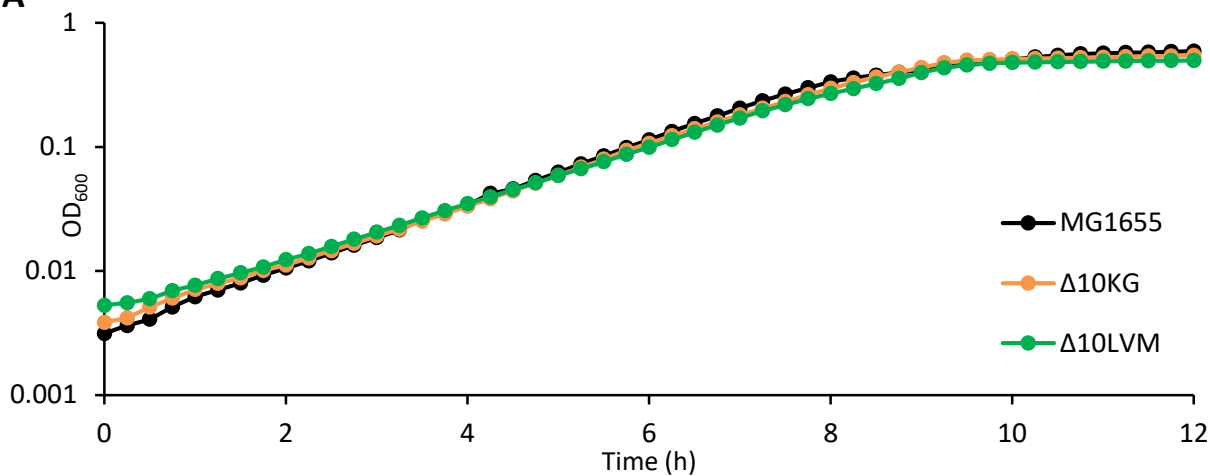**B**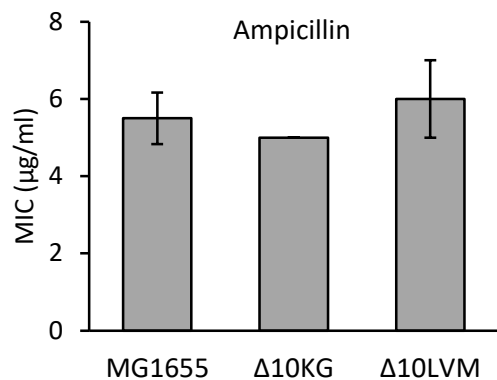**C**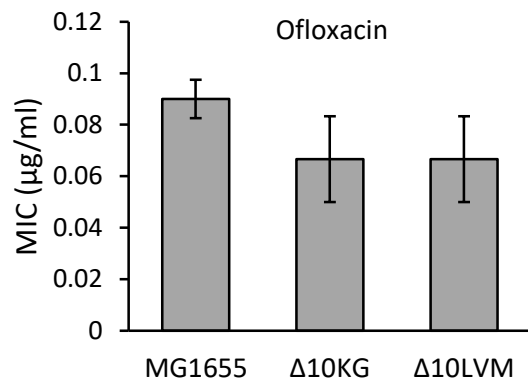**D**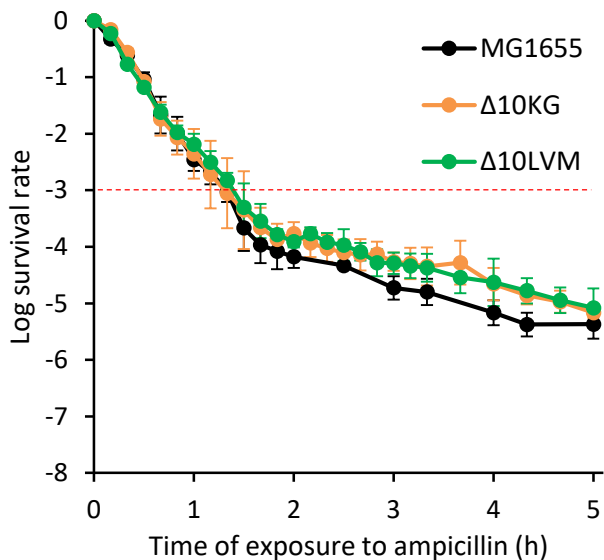**E**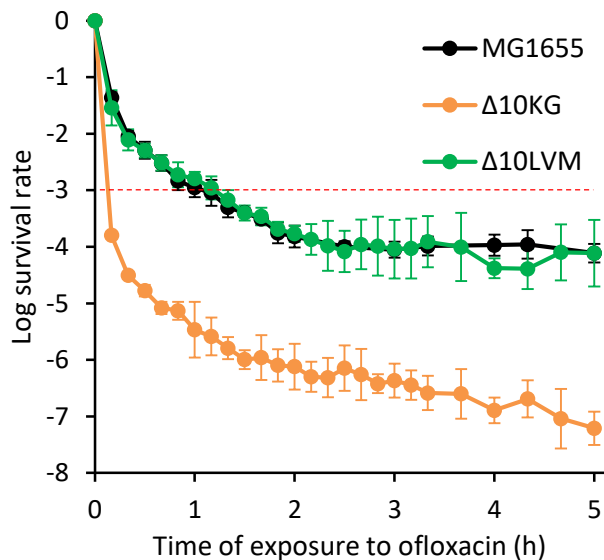

Supplement: FIG S1 [file mbo003183929sf1.pdf]

**A**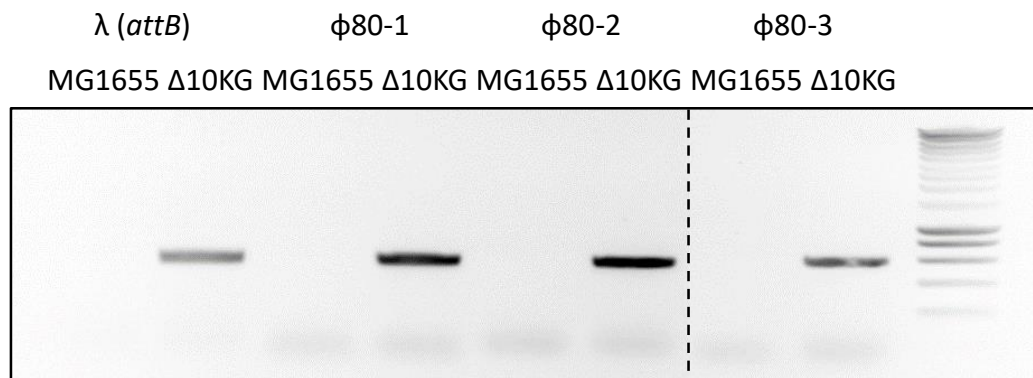**B**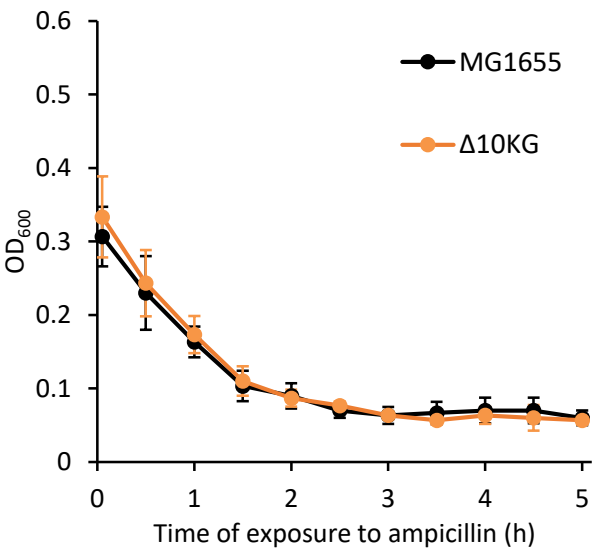**C**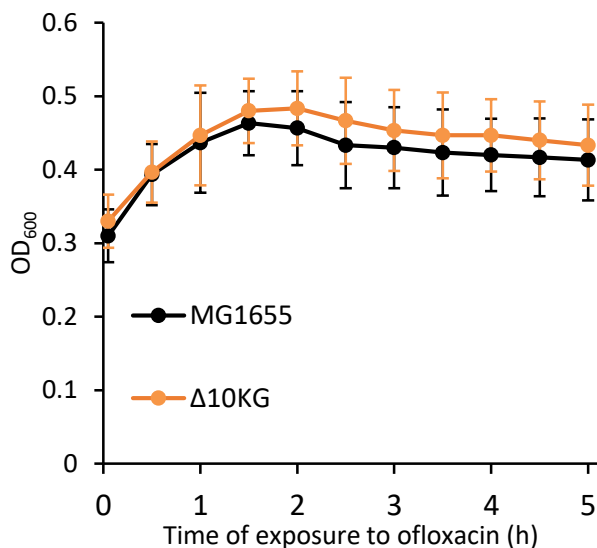

Supplement: FIG S2 [file mbo003183929sf2.pdf]

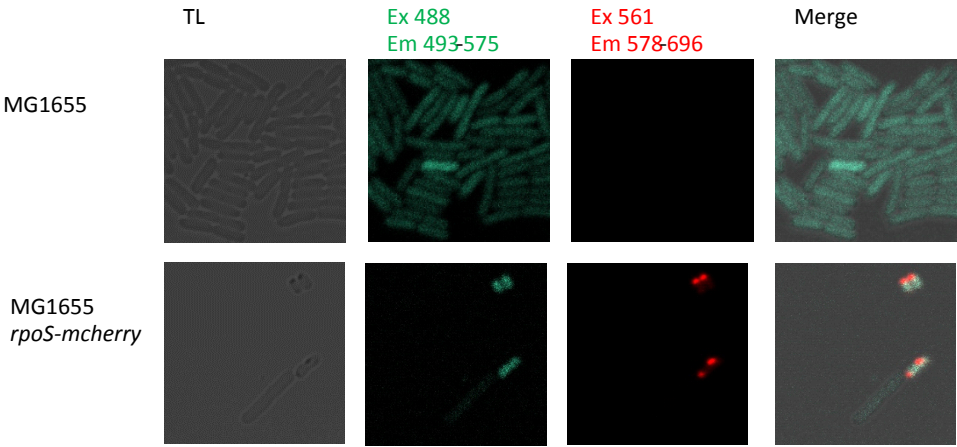

Supplement: FIG S3 [file mbo003183929sf3.pdf]

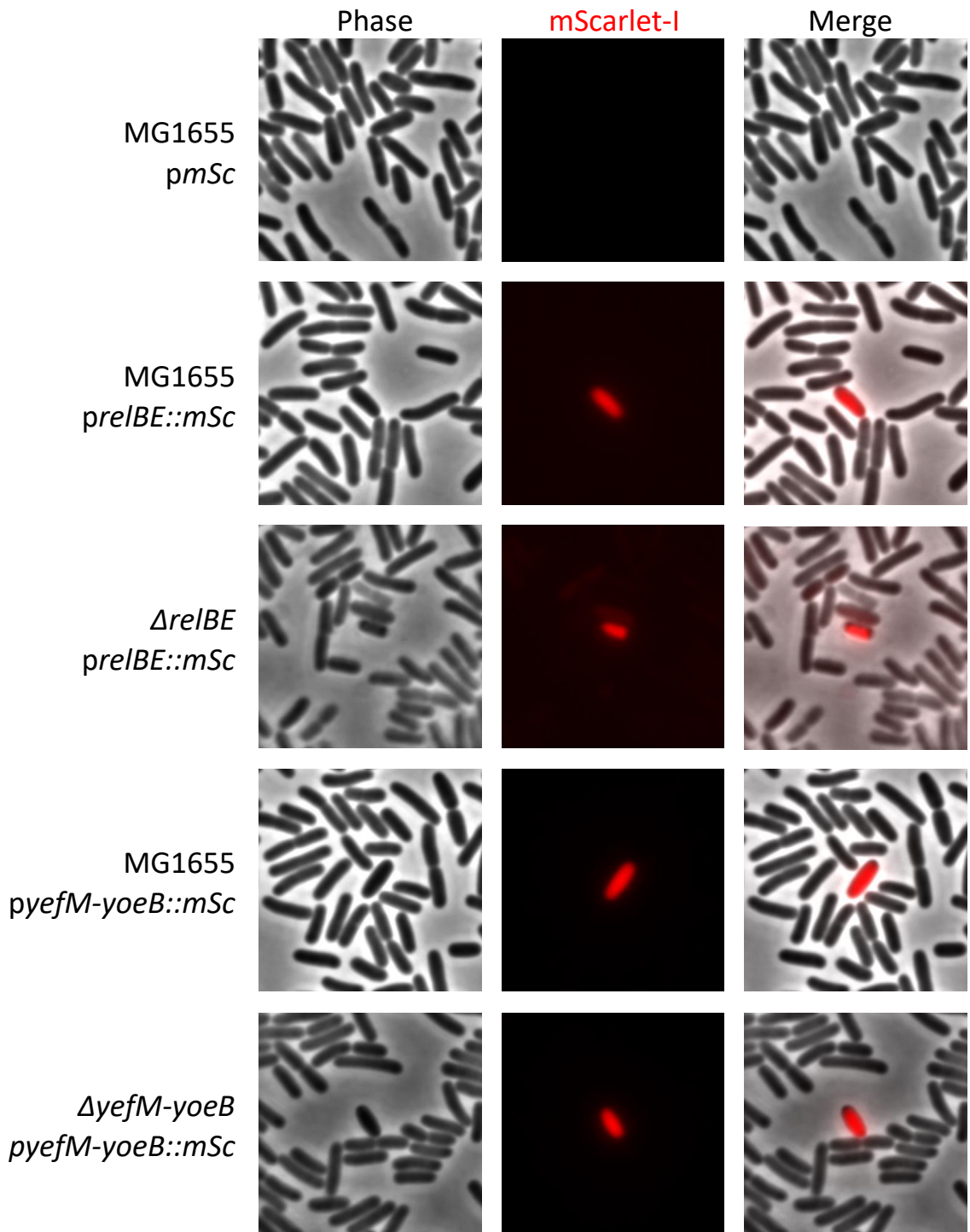

Supplement: FIG S4 [file mbo003183929sf4.pdf]
