# Supplementary material for: Reassessing the Role of Type II Toxin-Antitoxin Systems in Formation of Escherichia coli Type II Persister Cells
Source: mBio. 2018 Jun 12;9(3):e00640-18. doi: 10.1128/mBio.00640-18 (PMC6016239; doi:10.1128/mBio.00640-18)
Supplement: TABLE S1 [file mbo003183929st1.pdf]

Table S1: Genetic rearrangements and polymorphisms identified in TA deletion mutants

Coordinate	MG1655	*Δ10LVM*	*Δ5KG*	*Δ7KG*	*Δ8KG*	*ΔK9G*	*Δ10KG*	Description
**1340001**								Silent SNP in *pgpB* G→T
**4474834**								Mutation 27 bp before *tabA*
**4549192**								SNP in *fimH* T→G Thr→Asp
**807327**								λ prophage insertion
**1310570**								φ80-1 prophage insertion
**3192087**								φ80-2 prophage insertion
**2052027**								φ80-3 prophage insertion
**3801371**								Insertion in *waaJ* (T)
**677815-687900**								10 kbp deletion
**3038242**								SNP in *dsbC* G→C Gly→Ala
