# Supplementary material for: Reassessing the Role of Type II Toxin-Antitoxin Systems in Formation of Escherichia coli Type II Persister Cells
Source: mBio. 2018 Jun 12;9(3):e00640-18. doi: 10.1128/mBio.00640-18 (PMC6016239; doi:10.1128/mBio.00640-18)
Supplement: TABLE S2 [file mbo003183929st2.pdf]

Table S2: LFQ-MS analysis of Δ10 strains

	MG1655	Δ10KG	Δ10LVM
**MazE**	24.9	30.9	ND
**MazG**	26.7	30	ND
**GltI**	32	ND	30.9
**GltL**	27.6	ND	28.4
**RihA**	30.7	ND	30.8
YlaC	29.4	26.8	29.5
CobS	28.8	28.3	24.4
**TabA**	28.6	28.8	23.1
