# Supplementary material for: Reassessing the Role of Type II Toxin-Antitoxin Systems in Formation of Escherichia coli Type II Persister Cells
Source: mBio. 2018 Jun 12;9(3):e00640-18. doi: 10.1128/mBio.00640-18 (PMC6016239; doi:10.1128/mBio.00640-18)
Supplement: TABLE S3 [file mbo003183929st3.pdf]

Table S3: Strains and plasmids used in this study

Strain	Genotype	Reference
MG1655	*E. coli* K-12 F^-, λ^-, *ilvG*, *rfb-50*, *rph-1*	(1)
SC301467 (Δ5KG)	MG1655 *ΔmazF ΔchpSB ΔrelBEF ΔdinJ-yafQ ΔyefM-yoeB*	(2)
MGJ598 (Δ7KG)	Δ5KG *ΔhigBA ΔprlF-yhaV*	(2)
MGJ5987 (Δ8KG)	Δ7KG *ΔyafNO*	(2)
MGJ5987 (Δ9KG)	Δ8KG *ΔmqsRA*	(2)
MGJ5987 (Δ10KG)	Δ9KG *ΔhicAB*	(2)
LVM100 (Δ5LVM)	MG1655 *ΔchpSB ΔmazEF ΔrelBE ΔyefM-yoeB ΔdinJ-yafQ*	(3)
Δ10LVM	LVM100 *ΔyafNO ΔprlF-yhaV ΔhicAB ΔhigBA ΔmqsRA*	(4)
rpoS-mcherry	MG1655 *rpoS-mCherry::frt*	(5)
yoeB::gfp	MG1655 *yoeB::GFP::frt*	(5)
relE::gfp	MG1655 *relE::GFP::frt*	(5)
**Plasmid**	**Properties**	**Reference**
pET*gfpmut2*	pET41a p*tac::gfpmut2-AGGAGG(3)-CAT-trpterminator*	(6)
pBeloBAC11	*cat ori2(F) repE parABC cos lacZα*	NEB
p*mSc*	pBeloBAC11 *Δ(lacZα-cos)*::(*λt1ter-mScarlet-I-T7TE-luxIA*)	This study
p*yefMyoeB::mSc*	pBeloBAC11 *Δ(lacZα-cos)*::(*λt1ter-*p*yefMyoeB::mScarlet-I-T7TE-luxIA*)	This study
p*relBE::mSc*	pBeloBAC11 *Δ(lacZα-cos)*::(*λt1*-p*relBE*::*mScarlet-I-T7TE-luxIA*)	This study

References cited in Table S3

1. Blattner FR, Plunkett G, 3rd, Bloch CA, Perna NT, Burland V, Riley M, Collado-Vides J, Glasner JD, Rode CK, Mayhew GF, Gregor J, Davis NW, Kirkpatrick HA, Goeden MA, Rose DJ, Mau B, Shao Y. 1997. The complete genome sequence of Escherichia coli K-12. Science 277:1453-1462.

2. Maisonneuve E, Shakespeare LJ, Jorgensen MG, Gerdes K. 2011. Bacterial persistence by RNA endonucleases. Proc Natl Acad Sci U S A 108:13206-13211.

3. Tsilibaris V, Maenhaut-Michel G, Mine N, Van Melderen L. 2007. What is the benefit to Escherichia coli of having multiple toxin-antitoxin systems in its genome? J Bacteriol 189:6101-6108.

4. Goeders N, Dreze PL, Van Melderen L. 2013. Relaxed cleavage specificity within the RelE toxin family. J Bacteriol 195:2541-2549.

5. Maisonneuve E, Castro-Camargo M, Gerdes K. 2013. (p)ppGpp controls bacterial persistence by stochastic induction of toxin-antitoxin activity. Cell 154:1140-1150.

6. Luidalepp H, Joers A, Kaldalu N, Tenson T. 2011. Age of inoculum strongly influences persister frequency and can mask effects of mutations implicated in altered persistence. J Bacteriol 193:3598-3605.
