# Supplementary material for: Reassessing the Role of Type II Toxin-Antitoxin Systems in Formation of Escherichia coli Type II Persister Cells
Source: mBio. 2018 Jun 12;9(3):e00640-18. doi: 10.1128/mBio.00640-18 (PMC6016239; doi:10.1128/mBio.00640-18)
Supplement: TABLE S4 [file mbo003183929st4.pdf]

Table S4 : Primers used in this study

AvrII-relB F	CCCGCCTAGGCCAGTAAGATGATGGCCGG
NsiI-relB R	CCCCATGCATGTCTTACACCTCTTGTAATTACAAATGTCA
AvrII-yefM F	CCCGCCTAGGATTTTATGGCAATGGATTTGTTTAATGAATGAGC
NsiI-yefM R	CCGCATGCATGTCAATCTCCTCTTTTGTACAGT
ybhC (lambda) F	CGGTTTGATCAGAAGGACGTT
lambda R	GCTCTCGGAATATCAATGAAGG
kch (phi80-1) F	ACTGTACCTCAGGAAAGGTCA
glgS (phi80-2) F	CATCAAACGTCAATGGGGTGC
yeeJ (phi80-3) F	CATTACACGACTGACGCTGGTT
phi80 R	CTGAAAACAGCAAGTGGTCGC
