# Supplementary material for: Reassessing the Role of Type II Toxin-Antitoxin Systems in Formation of Escherichia coli Type II Persister Cells
Source: mBio. 2018 Jun 12;9(3):e00640-18. doi: 10.1128/mBio.00640-18 (PMC6016239; doi:10.1128/mBio.00640-18)
Supplement: TEXT S1 [file mbo003183929s1.pdf]

Supplementary Materials and Methods

Growth curves. Cultures were grown in 12-wells microtiter plates containing 2 ml MOPS-glucose medium per well. Overnight cultures were diluted so as to inoculate cultures at OD_{600} 0.01. Microtiter plates were then incubated at 37°C with stirring in a SpectraMax i3 detection platform. Turbidity (OD_{600}) was measured every 15 min during 24 h.

MIC measurements. Minimal inhibitory concentration (MIC) measurements were performed using the agar dilution method as described in (1). Briefly, overnight cultures were spotted on LB plates containing 2-fold increasing concentrations of ampicillin or ofloxacin. The MIC value was defined as the lowest concentration of antibiotic inhibiting growth.

Confirmation of phage insertions by PCR. Lysogenization by 4 prophages was confirmed by polymerase chain reaction (PCR) using Q5 DNA polymerase (New England Biolabs) according to the manufacturer's protocol. Single colonies grown on LB-agar were boiled in water and the junction between each phage and its specific insertion site were amplified using primers listed in Table S4.

Label-Free Quantification analysis. Triplicate cultures of MG1655, Δ10KG and Δ10LVM were grown overnight in autoclaved LB medium and diluted 1,000 fold. Cultures were sampled in exponential phase after 4h of growth (OD_{600} ~1.9), washed with PBS and flash-frozen in liquid nitrogen before storage at 80°C. Cell pellets were lysed with heat and sonication in a SDS lysis buffer. Extracted proteins were precipitated with methanol-chloroform and digested in denaturing conditions with Lys-C and trypsin. Peptides were desalted and injected to a nano-LC MS/MS system operated in a data-dependent acquisition mode. Raw data were analyzed using MaxQuant software (2) v. 1.5.6.5. Summed peptide peak areas (protein intensities) were normalized using the MaxLFQ algorithm (3). Perseus software (4) v. 1.5.8.5 was used for further data analysis.

Contaminants and proteins identified only by a modification site were removed from the data matrix and the LFQ intensity values were $\log_2$ transformed (5). Normalized LFQ intensity values were used as the quantitative measurement of protein abundance. Statistically significant proteins were identified by one-way ANOVA (Benjamini-Hochberg FDR <0.05) followed by Tukey-Kramer post-hoc test (p<0.05 threshold) using Real Statistics Resource Pack for Excel 2010 (Release 5.1).

References

1. **Wiegand I, Hilpert K, Hancock RE.** 2008. Agar and broth dilution methods to determine the minimal inhibitory concentration (MIC) of antimicrobial substances. Nat Protoc **3:**163-175.
2. **Cox J, Mann M.** 2008. MaxQuant enables high peptide identification rates, individualized p.p.b.-range mass accuracies and proteome-wide protein quantification. Nat Biotechnol **26:**1367-1372.
3. **Cox J, Hein MY, Luber CA, Paron I, Nagaraj N, Mann M.** 2014. Accurate proteome-wide label-free quantification by delayed normalization and maximal peptide ratio extraction, termed MaxLFQ. Mol Cell Proteomics **13:**2513-2526.
4. **Tyanova S, Temu T, Sinitcyn P, Carlson A, Hein MY, Geiger T, Mann M, Cox J.** 2016. The Perseus computational platform for comprehensive analysis of (prote)omics data. Nat Methods **13:**731-740.
5. **Quackenbush J.** 2002. Microarray data normalization and transformation. Nat Genet **32 Suppl:**496-501.
